# Influence of grid resolution of large‐eddy simulations on foehn‐cold pool interaction

**DOI:** 10.1002/qj.4281

**Published:** 2022-05-10

**Authors:** L. Umek, A. Gohm, M. Haid, H. C. Ward, M. W. Rotach

**Affiliations:** ^1^ Department of Atmospheric and Cryospheric Sciences University of Innsbruck Innsbruck Austria

**Keywords:** cold‐air pool, complex terrain, foehn, grey zone of turbulence, model sensitivity, PIANO, turbulent erosion

## Abstract

Numerical simulations are performed to assess the influence of horizontal and vertical model resolution on the turbulent erosion of a cold‐air pool (CAP) by foehn winds in an Alpine valley near Innsbruck, Austria. Strong wind shear in the transition zone from the CAP to the overlying foehn generates turbulence by shear‐flow instability and contributes to the CAP erosion. The sensitivity of this process to grid resolution in the “grey zone” of turbulence is studied with the Weather Research and Forecasting model in large‐eddy simulation (LES) mode with a horizontal grid spacing of 200, 40, and 13.33 m and in mesoscale mode with a grid spacing of 1 km. Moreover, two different vertical resolutions are tested. The mesoscale simulation exhibits deficiencies in the CAP development and is neither able to resolve nor parametrize the effect of Kelvin–Helmholtz (K–H) instability. In contrast, the LES with the coarsest horizontal grid spacing begins to explicitly permit K–H instability, albeit individual K–H waves are not completely resolved, and thereby greatly improves the stability and wind profile of the foehn. Refining the LES grid spacing leads to a more explicit and realistic representation of turbulence, but surprisingly has little impact on mean quantities. An increase in the vertical resolution shows the greatest benefit in the turbulent upper part of the foehn jet, whereas an increase in the horizontal resolution improves the turbulence characteristics, especially at the foehn–CAP interface. However, spectral analysis indicates that even a horizontal grid spacing of 40 m does not fully capture the energy cascade in the inertial subrange. Eddies remain too large and foehn–CAP interaction is too vigorous compared with the simulation with 13.33 m grid spacing. Nevertheless, results illustrate the potential benefit of an 𝒪(100 m) model resolution for improving numerical weather predictions in complex terrain.

## Graphical abstract

Shear‐flow instability is found to be an important process of cold‐air pool erosion by foehn winds. Compared with a large‐eddy simulation with a horizontal mesh size of 13 m, the effect of this turbulent process is much better represented at a 200 m grid size (although not fully resolved) than parametrized at a kilometre resolution. It is concluded that 𝒪(100 m) model resolution will improve numerical weather predictions in complex terrain.

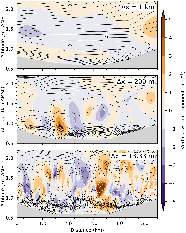

## 1 INTRODUCTION

Numerical weather prediction (NWP) based on hectometre model grid spacing will become realistic in the near future and is envisioned to improve the wind forecast over complex terrain for various practical applications (e.g., Olson *et al*., 2019; Wagner *et al*., 2019; Wong *et al*., 2013). However, this resolution lies in the “grey zone” of turbulence, also known as “terra incognita” (Wyngaard, 2004), which poses various challenges for mesoscale and microscale modelling (Chow *et al*., 2019; Honnert *et al*., 2020). For example, it is known that the structure of downslope wind storms is sensitive to the model resolution (e.g., Chan *et al*., 2021; Orr *et al*., 2021). However, systematic studies on the benefit of increased resolution over the full kilometre to decametre range are scarce (Rai *et al*., 2019). This is especially true for strong winds over complex terrain. In an attempt to fill in this gap, we extend an earlier case study (Umek *et al*., 2021) to assess the influence of the horizontal and vertical resolution of large‐eddy simulations (LES) on the structure and development of foehn winds in an Alpine valley near Innsbruck, Austria. More specifically, the simulated interaction of the foehn with a cold‐air pool (CAP) in a valley is investigated with a focus on CAP erosion due to turbulent mixing with potentially warmer foehn air. Hence, we will clarify to what extent Kelvin–Helmholtz (K–H) instability at the foehn–CAP interface is explicitly resolved by different LES model resolutions and how well it is parametrized in a mesoscale model. For this purpose, the various LES conducted in this study are compared with a mesoscale simulation with a mesh size of 1 km, which is a typical resolution of today's operational high‐resolution limited‐area NWP models. In the broadest sense, our study helps to clarify the benefits of increasing the resolution of NWP models in the future.

**FIGURE 1:**
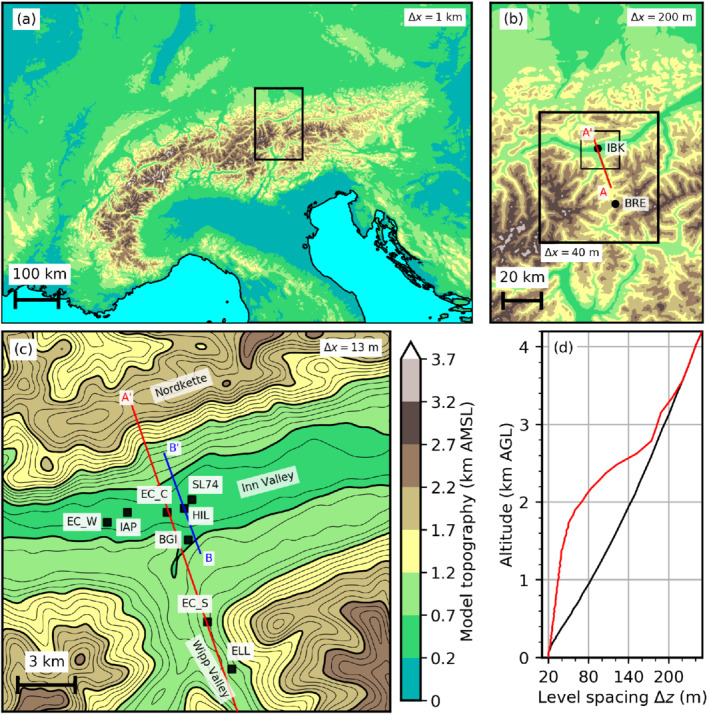
(a) Domain of the mesoscale simulation (DX1) covering the Alpine region. WRF model topography is illustrated by colour contours with a 500 m interval. The black rectangle indicates the location of the outermost large‐eddy simulation (LES) domain shown in (b). (b) Domain of the LES with a horizontal grid spacing of Δx=200 m (DX200 and DX200R). Two black rectangles indicate the location of two one‐way nested domains with Δx=40 m (DX40 and DX40R, thick line) and Δx=13.33 m (DX13, thin line), respectively. The red line shows the orientation of the vertical transect AA${}^{\prime }$ through the Wipp Valley. (c) Domain of DX13. Thick (thin) black contour lines illustrate the model topography with an interval of 500 m (100 m). The blue line indicates the orientation of the vertical transect BB${}^{\prime }$ across the Inn Valley. The red line illustrates the downstream part of the vertical transect AA${}^{\prime }$ shown in (b). Black markers indicate points of interest mentioned in the text. (d) Vertical model level spacing for the simulations with the default setup (black line) as used in Umek *et al.* (2021) and the refined model level spacing (red line). The latter is used in the simulations DX200R and DX40R [Colour figure can be viewed at wileyonlinelibrary.com]

Innsbruck (IBK in Figure 1b), the target area of this study, is located at about 570 m above mean sea level (AMSL) in the eastern European Alps where the west–east‐aligned Inn Valley joins the south–north‐orientated Wipp Valley (Figure 1). The latter connects Innsbruck with the lowest pass across the main Alpine crest, the Brenner Pass (BRE in Figure 1b; 1371 m AMSL), and is very prone to south foehn. The large‐scale forcing leading to south foehn in the Wipp Valley was extensively investigated during the Mesoscale Alpine Programme (Bougeault *et al*., 2001) and resulted in a number of observational and numerical studies (e.g., Flamant *et al*., 2002; Gohm and Mayr, 2004; Gohm *et al*., 2004; Mayr and Armi, 2008; Mayr *et al*., 2007; Zängl, 2003; Zängl and Gohm, 2006; Zängl *et al*., 2004). For a more complete literature review, the reader is referred to Umek *et al*. (2021).

Recently, the research project “Penetration and Interruption of Alpine Foehn” (PIANO) shifted the scientific focus towards smaller‐scale processes, namely the interaction of the south foehn emanating from the Wipp Valley with a CAP in the Inn Valley near Innsbruck. This CAP can prevent foehn air from reaching the Inn Valley floor. Therefore, foehn can only break through at the Inn Valley floor once the CAP has been removed. Within the PIANO project, the foehn–CAP interaction and erosion have been studied during seven intensive observation periods (IOPs) in fall and winter 2017. All except the first IOP were south foehn events. During two IOPs (IOP5 and IOP6) the CAP in the Inn Valley was not eroded, hence preventing a foehn breakthrough at the Inn Valley floor while south foehn was present in the Wipp Valley. During other IOPs (e.g., IOP3), a foehn breakthrough was observed only near the city centre of Innsbruck, whereas the CAP prevailed in other locations. The reader is referred to Haid *et al*. (2020; 2022) and Muschinski *et al*. (2020) for a comprehensive observational overview of the PIANO IOPs.

Haid *et al*. (2020) mention three potential processes of CAP removal: (a) turbulent erosion of the CAP from above due to wind shear; (b) diabatic heating of the CAP from the bottom by the surface sensible heat flux; or (c) a dynamic displacement of the CAP by the foehn air. However, there is disagreement in the literature about which of these processes is the most important (e.g., Flamant *et al*., 2006; Fritts *et al*., 2010; Jaubert *et al*., 2005; Lareau and Horel, 2015; Mayr and Armi, 2010; Strauss *et al*., 2016; Sheridan, 2019; Zhong *et al*.; 2003; 2001). Hence, it appears that the contribution of each of these processes is location, time, and case dependent. Turbulent CAP erosion from the top downward is often caused by shear‐induced turbulence at the foehn–CAP interface (Flamant *et al*., 2006; Nater *et al*., 1979; Tollinger *et al*., 2019). K–H waves (often also called K–H billows; e.g., Blumen *et al*., 2001; Fritts *et al*., 2012; 2020; Palmer *et al*., 1996) were explicitly observed at the foehn–CAP interface by Haid *et al*. (2020; 2022) based on coordinated scans with multiple Doppler wind lidars during various IOPs of the PIANO field campaign. Moreover, an LES case study of PIANO IOP2 also showed K–H instability at the foehn–CAP interface (Umek *et al*., 2021). Haid *et al*. (2020) and Umek *et al*. (2021) found that turbulent erosion of the CAP played a crucial role in facilitating foehn penetration to the Inn Valley bottom. Umek *et al*. (2021) showed for IOP2 that an LES with a horizontal grid spacing of Δx=40 m is closer to reality in forming and maintaining the CAP in the valley during night‐time than a mesoscale simulation with Δx=1 km. However, turbulent erosion during night‐time is too strong in the LES, leading to a shallower CAP in the simulation than in the observations. It was hypothesized that individual eddies may remain too large in the simulation, resulting in a too vigorous turbulent mixing of CAP and foehn air (Umek *et al*., 2021). However, a systematic study to test this hypothesis is still missing.

Operational mesoscale NWP models with a horizontal grid spacing of about Δx=1 km partly struggle to form and maintain a CAP in complex terrain (Crosman and Horel, 2017; Sandner, 2020; Wilhelm, 2012). Moreover, the simulated near‐surface potential temperature of the foehn jet in mesoscale NWP models exhibits a cold bias compared with observations (Sandner, 2020). This cold bias subsequently influences the simulation of the foehn–CAP interaction. Hence, a too early foehn penetration to the valley bottom is often forecasted in these NWP models (Sandner, 2020). Additionally, meso‐γ‐scale motions like K–H waves resulting from shear‐flow instability can lead to pulsations in wind speed and are barely resolved in mesoscale NWP simulations (Belušić *et al*., 2007; Tollinger *et al*., 2019). However, increasing computational power and advances in numerical modelling over recent years facilitate the move towards simulations with a horizontal grid spacing of 𝒪(100 m) for real‐case simulations. Nevertheless, refining the horizontal and vertical grid spacing from mesoscale simulations to LES leads across the “grey continuum”, which describes the transition from the parametrization of various physical processes (e.g., convection and turbulence) towards their explicit resolution (Chow *et al*., 2019). Therefore, these physical processes are only partially resolved in the “grey continuum”, and each process exhibits its own individual “grey zone” determined by its intrinsic length and/or time scale (Chow *et al*., 2019; Muñoz‐Esparza *et al*., 2014). More specifically, in the context of this study, the simulation of turbulent motions within the foehn jet and within the stably stratified CAP includes at least two “grey zones”. Though a particular mesh size might lead to the major part of turbulence being explicitly resolved within the foehn jet, a significantly finer grid spacing would be required to resolve the weaker and intermittent turbulence inside the CAP (Beare *et al*., 2006; Cuxart, 2015).

Numerical sensitivity studies across multiple “grey zones” have become popular in recent years, but most often are focused on single aspects. Wagner *et al*. (2014) investigated the development of a daytime planetary boundary layer (PBL) and found that unresolved topography exhibits a larger influence than unresolved turbulent processes in idealized simulations. Hughes *et al*. (2015) and Duine and De Wekker (2017) noted that, with coarser grid spacings, CAPs are poorly simulated due to unresolved topography. Vosper *et al*. (2013) and Hughes *et al*. (2015) showed that the vertical model level spacing influences the simulated CAP formation. In the latter study, this was mostly attributed to differences in cloud cover. Beare and Macvean (2004) investigated the influence of the horizontal resolution on the stable boundary layer characteristics down to a grid spacing of 2 m and Beare *et al*. (2006) the influence of the chosen subgrid‐scale (SGS) turbulence model on stable boundary layers. Liu *et al*. (2020) studied the performance of several PBL parametrizations and SGS turbulence models across the grey zone. Additionally, several studies investigated the influence of grid spacings across the grey zone on the formation of clouds and precipitation (e.g., Barthlott and Hoose, 2015, Boutle *et al*., 2014, Heim *et al*., 2020, Langhans *et al*., 2012, Singh *et al*., 2021, Stevens *et al*., 2020). To our best knowledge, studies that investigate the influence of model grid spacing in the grey zone on the simulation of downslope wind storms, such as foehn, are limited. Zängl and Gohm (2006) simulated the foehn in the Wipp Valley with the MM5 model and a horizontal grid spacing of about 500 m and showed that this simulation was more realistic than one with a horizontal mesh size of 800 m. Zängl *et al*. (2008) also used the MM5 model to investigate the influence of different model level settings on the foehn flow. They found that a lower height of the lowest model level above ground seems to improve the wind field but not necessarily the temperature field. Pattantyus *et al*. (2011) found that a higher number of vertical model levels in the boundary layer improved the representation of the gustiness of a downslope wind storm near Las Vegas using the Weather Research and Forecasting (WRF) model. Orr *et al*. (2021) also used the WRF model to simulate the foehn over the Antarctic Peninsula and its interaction with a CAP with horizontal grid spacings of 4, 1.5, and 0.5 km. They found that hydraulic jumps and leeside warming were quite insensitive to resolution, with no evidence of convergence of the results at higher resolution. However, individual foehn jets showed considerable dependence on the grid spacing. None of these studies investigated the model sensitivity on horizontal grid spacing in the decametre range.

The overall aim of this study is to examine the sensitivity of the development and structure of the foehn and the CAP on the horizontal and vertical model grid resolution. More precisely, we want to assess whether the grid resolution in the grey zone of turbulence and beyond has an impact on

- the vertical structure of the foehn jet in the Wipp Valley,
- the turbulent erosion of the CAP in the Inn Valley, and
- the foehn breakthrough at the valley floor.

For this purpose, this work extends the study of Umek *et al*. (2021) through additional simulations for the same foehn event (PIANO IOP2) with even higher horizontal and vertical grid resolutions. Section 2describes the model set‐up and relevant observations. An overview of the foehn evolution and breakthrough in the various simulations is given in Section 3.1. The influence of the grid resolution on the foehn jet in the Wipp Valley is assessed in Section 3.2 and on the foehn–CAP interaction in the Inn Valley in Section 3.3. Results are discussed in Section 4, and conclusions are drawn in Section 5.

## 2 MODEL AND OBSERVATIONS

This work extends the study of Umek *et al*. (2021) and, therefore, uses a similar set‐up of the WRF model, version 4.1 (Skamarock *et al*., 2019). Details on the model set‐up can be found in Umek *et al*. (2021) and Umek (2022). An overview of the simulations is given in Table 1. A mesoscale simulation with a horizontal grid spacing of Δx=1 km (DX1) covers the greater Alpine region (Figure 1a). DX1 is initialized at 1200 UTC November 3, 2017, and uses the operational high‐resolution analysis of the European Centre for Medium‐Range Weather Forecasts with a six‐hourly interval as initial and boundary conditions. Moreover, observational nudging is active until lead time +6 hr in order to provide realistic initial conditions for the LES, see Umek *et al*. (2021, appendix A).

**TABLE 1:** Short description of the numerical Weather Research and Forecasting simulations. See text for further details

Simulationn	Initialization time	Δx	Vertical levels	Boundary conditions and nesting
DX1	1200 UTC November 3, 2017	1 km	80	ECMWF high‐resolution analysis data, 6‐hr interval
DX200	1800 UTC November 3, 2017	200 m	80	Offline nesting in DX1 via ndown, 30‐min interval
DX40	1800 UTC November 3, 2017	40 m	80	One‐way online nesting in DX200
DX13	1800 UTC November 3, 2017	13.33 m	80	One‐way online nesting in DX40
DX200R	1800 UTC November 3, 2017	200 m	110	Offline nesting in DX1 via ndown, 30‐min interval
DX40R	1800 UTC November 3, 2017	40 m	110	One‐way online nesting in DX200R

ECMWF: European Centre for Medium‐Range Weather Forecasts.

Output data of DX1 at a 30‐min interval is used with the ndown‐tool of the WRF software framework to calculate initial and boundary conditions for subsequent stand‐alone (i.e., offline nested) LES. The latter are initialized at 1800 UTC November 3. Two sets of LES are used to investigate the sensitivity to the horizontal and vertical grid spacing. Both are based on the LES model configuration of Umek *et al*. (2021), which includes two one‐way, online nested domains with a horizontal grid spacing of Δx=200 m (DX200) and Δx=40 m (DX40) to cover the Inn Valley and Wipp Valley region (Figure 1b). The first set, however, uses a third online nested domain with Δx=13.33 m (DX13) and 1,510×1,480 grid points to evaluate the impact of even higher horizontal grid resolution. This domain focuses on the Wipp Valley exit region and the Inn Valley around Innsbruck (Figure 1c). The large computational costs associated with such a small grid size strongly limits the domain size. Nevertheless, the domain is, with about 20×20 km${}^{2}$, large enough to include a fetch region of several kilometres from the southern boundary towards the target region where the foehn jet interacts with the CAP in the Inn Valley (Figure 1c). Similar to Liu *et al*. (2020), we do not use a cell perturbation method (Muñoz‐Esparza *et al*., 2014) to spin up turbulence. The potential impact of this approach will be assessed in Section 4. Static data and initial conditions are interpolated from domain DX40 to domain DX13. The latter uses a time step of 0.0833 s. To ensure numerical stability, the namelist variable epssm has been set to a value of 0.9 for the LES simulations. Moreover, the namelist variables emdiv and smdiv are set to a value of 0.02 and 0.2 for the LES domains with 40 m and 13 m horizontal grid spacing, respectively. The default values for these namelist variables (0.1 and 0.01, respectively) are used for the outermost LES domain with a horizontal grid spacing of 200 m. Further details on the model set‐up can be found in Umek *et al*. (2021). All three domains use 80 vertical levels, with the lowest model level at about 10 m above ground level (AGL) and a vertical level spacing of Δz=20 m near the surface. Δz is stretched with height and reaches 140 m at about 2 km AGL (black line in Figure 1d). In the area of Innsbruck, this height corresponds to about 2,500 m AMSL, which is close to the surrounding crest height (Figure 1b).

The second set of LES is based on two one‐way nested domains with a horizontal grid spacing of Δx=200 m and Δx=40 m and an increased number of vertical levels (110 in total), resulting in a refined model level spacing. These simulations are denoted DX200R and DX40R, respectively. The level spacing is approximately half of that used in the original model set‐up in the layer up to 2 km AGL (red line in Figure 1d). From 2 to 3.5 km AGL the model level spacing fades towards the original set‐up. For the lowest four model levels a vertical refinement was not possible due to numerical instabilities at the surface in complex terrain. These levels are located at about 10, 30, 50, and 75 m AGL and are therefore identical in all LESs (see lowest part of Figure 1d). A third domain with Δx=13.33 m and a refined vertical level spacing was not possible due to enormous computational costs.

Observational data collected during the PIANO field campaign are used to evaluate the numerical simulations. This includes data from an automatic weather station operated by the Department of Atmospheric and Cryospheric Sciences of the University of Innsbruck in the lower Wipp Valley close to the village of Ellbögen (ELL in Figure 1c; 1,080 m AMSL) and data from an eddy‐covariance (EC) station operated on the rooftop of the Department of Atmospheric and Cryospheric Sciences in the centre of Innsbruck (EC_C in Figure 1c; 621 m AMSL) as part of the Innsbruck Atmospheric Observatory (Karl *et al*., 2020). EC_C sampled data at 10 Hz, and turbulence kinetic energy (TKE) is derived for individual 30‐min intervals. Additionally, data from a radiosonde ascent conducted at Innsbruck Airport (IAP in Figure 1c; 578 m AMSL) is used in this study. Four scanning Doppler wind lidars were operated on top of high buildings during PIANO (Haid *et al*., 2020). In this study, data of the SL74 lidar near the centre of Innsbruck (Figure 1c) are used to derive vertical profiles of the vertical velocity variance. This is only a small part of all observations available during the PIANO field campaign, and the reader is referred to Haid *et al*. (2020; 2022) and Muschinski *et al*. (2020) for a comprehensive overview. The PIANO data are available online (Gohm *et al*.,, 2021a; 2021b; 2021c; 2021d).

## 3 RESULTS

### 3.1 Overview of PIANO IOP2

This section provides a concise overview of the cold pool evolution and the foehn onset in the Wipp Valley and the region around Innsbruck from November 3 to 4, 2017, representing the first part of PIANO IOP2, based on observational data from EC_C and ELL (Figure 1c) and the numerical simulations. A detailed analysis of the event based on observations can be found in Haid *et al*. (2020). The reader is also referred to Umek *et al*. (2021) for a detailed evaluation of the performance of the LES with a horizontal grid spacing of 40 m (the simulation denoted DX40 herein), whereas in this article we focus on a comparison of simulations with various different grid resolutions.

**FIGURE 2:**
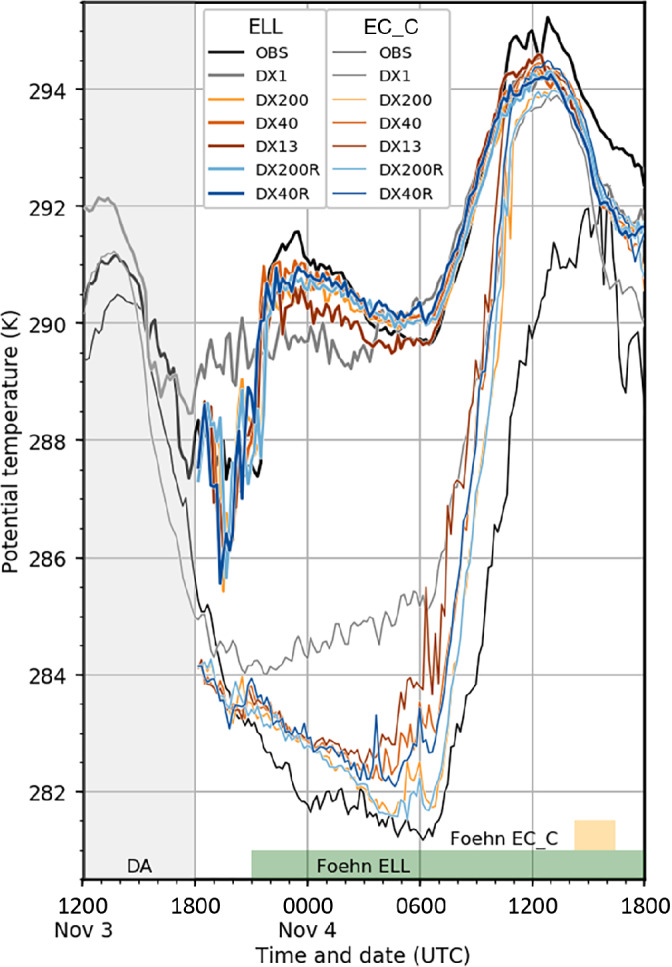
Time series of 10‐min averaged near‐surface potential temperature at Ellboegen (ELL; 1080 m AMSL) in the Wipp Valley (thick lines) and in the centre of Innsbruck (EC_C; 621 m AMSL) in the Inn Valley (thin lines) from 1200 UTC November 3, 2017, to 1800 UTC November 4, 2017 (see Figure 1 for locations). Sunrise (sunset) is at about 0600 UTC (1550 UTC) on November 4. Observations are shown in black and six different simulations in colour. Simulated potential temperatures represent values diagnosed at 2 m AGL. All LES are initialized at 1800 UTC November 3. The period with ongoing foehn at ELL is indicated by green shading, and a transient foehn breakthrough observed at EC_C (cf. Haid *et al.*, 2020) is indicated by yellow shading. The period of data assimilation (DA) through observation nudging is illustrated by grey shading [Colour figure can be viewed at wileyonlinelibrary.com]

After 2000 UTC November 3, south foehn was observed in the southern part of the north–south‐aligned Wipp Valley. The foehn reached the bottom of the Wipp Valley exit region about 2 hr later. At ELL, the observed foehn penetration was characterized by a sharp increase in potential temperature by 4 K to about 291 K (thick black line in Figure 2). All LES capture the abrupt onset of foehn in terms of time and magnitude of temperature increase (thick coloured lines in Figure 2). The mesoscale simulation DX1 exhibits a too early foehn onset at ELL and a cold bias of up to 2 K during night‐time after the observed foehn breakthrough (thick grey line in Figure 2).

Though observed near‐surface foehn temperatures in the Wipp Valley decreased only by about 1 K during the night, a strong nocturnal CAP formed in the west–east‐aligned Inn Valley with potential temperature at 0600 UTC being about 9 K lower than in the Wipp Valley. Overall, all LES reproduce the weak cooling of the near‐surface foehn air at ELL and the stronger nocturnal cooling in the Inn Valley reasonably well. In contrast, the mesoscale simulation DX1 exhibits an increase in potential temperature already after 2000 UTC November 3 at EC_C (thin lines in Figure 2). After 0300 UTC November 4, temporal variability of the simulated near‐surface potential temperature increases in most of the LES. This is caused by foehn air mixing with the rather shallow CAP and influencing the lowest model levels above the Inn Valley floor.

A foehn breakthrough in the Inn Valley is often indicated by a neutral to near‐neutral stratification in the lowest few hundred metres above the valley floor and diagnosed when potential temperature in the Wipp Valley exit region equals potential temperature observed at the Inn Valley bottom (e.g., at ELL and EC_C, respectively). After sunrise, the rapid increase in observed potential temperature in the Inn Valley led to a full foehn breakthrough in the west of Innsbruck in the afternoon of November 4 (not shown; see Haid *et al*. (2020) and Umek *et al*. (2021)). However, in the city centre the observed near‐surface potential temperature stayed about 1 K below the foehn temperature, resulting in a transient foehn breakthrough there (cf. EC_C and ELL during the period marked in yellow in Figure 2). All LES exhibit a similar temperature increase, which starts about 2–3 hr earlier than observed and ultimately results in a simulated foehn breakthrough in the city centre at about noon. The CAP erosion in DX1 already started during night‐time, however, the timing of the simulated foehn breakthrough in the city centre is about the same as in the LES.

In the afternoon of November 4, the simulated near‐surface potential foehn temperature decreases slightly more strongly than observed at ELL. A foehn interruption and CAP reformation in the Inn Valley is observed (i.e., strong drop in potential temperature at EC_C) while ongoing foehn is present at EC_C in all simulations (Figure 2). After 1500 UTC November 4, the DX1 simulation exhibits stronger cooling at EC_C than at ELL; however, wind speed and direction in DX1 still confirm the presence of south foehn at the Inn Valley bottom at that time (not shown). Hence, the foehn jet is more stably stratified in the Inn Valley in DX1 compared with the LES.

### 3.2 Stratification of foehn layer in the Wipp Valley

**FIGURE 3:**
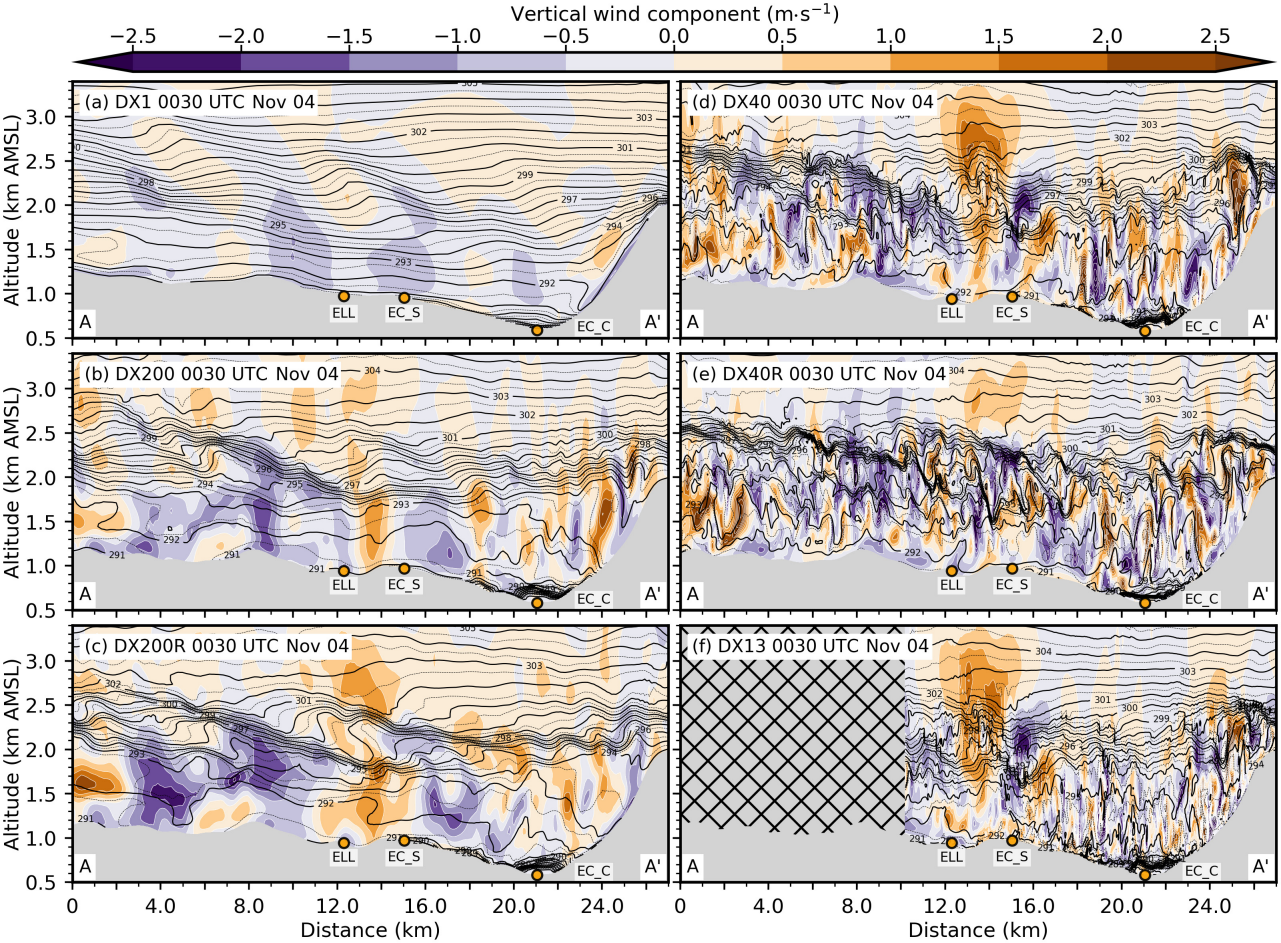
Vertical cross‐section from south to north along the Wipp Valley and across the Inn Valley (see transect AA${}^{\prime }$ in Figure 1b) of potential temperature and vertical wind component at 0030 UTC November 4, 2017 for (a) DX1, (b) DX200, (c) DX200R, (d) DX40, (e) DX40R, and (f) DX13. Flow direction of the foehn flow is from south (left) to north (right). Potential temperature is shown as black solid and dotted contour lines (0.5 K increments). Vertical wind component is illustrated as colour contours with 0.5 m·s${}^{-1}$ increments. Grey shading indicates the model topography and the hatched region in (f) is outside of the DX13 domain (see Figure 1b). Annotated orange dots show points of interest as in Figure 1c [Colour figure can be viewed at wileyonlinelibrary.com]

Figure 3 shows the instantaneous potential temperature and vertical wind component for all numerical simulations on a vertical transect along the Wipp Valley and across the Inn Valley (see ${\text{AA}}^{\prime }$ in Figure 1b) at 0030 UTC November 4. At this time, south foehn is well established in the Wipp Valley, and densely packed isentropes near EC_C illustrate the presence of a CAP in the Inn Valley (Figure 3). As already mentioned, this CAP is weaker in the mesoscale simulation DX1 than in the LES (Figure 3). In the latter, near‐surface potential temperature is about 2–3 K lower (Figure 2).

**FIGURE 4:**
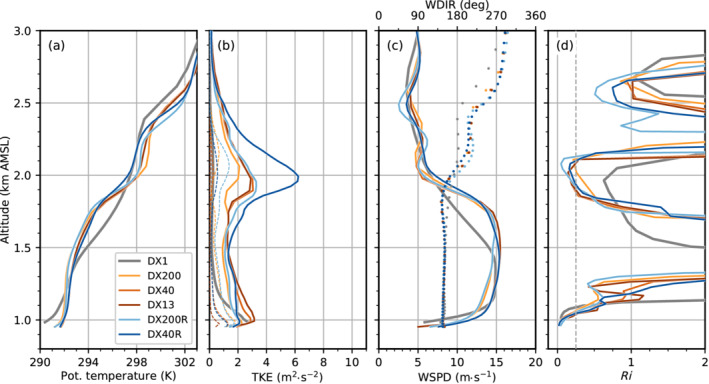
Vertical profiles of (a) potential temperature, (b) turbulence kinetic energy (TKE), (c) horizontal wind speed (WSPD, lines) and direction (WDIR, dots), and (d) Richardson number (Ri) at ELL in the Wipp Valley. Data are averaged from 2300 UTC November 3 to 0100 UTC November 4, 2017, for six different simulations (see legend). The mean total TKE is shown by thick solid lines, and the parametrized subgrid‐scale TKE by thin dashed lines. The resolved part of TKE can be inferred from the difference between the solid and dashed line of a corresponding simulation. The Richardson number in (d) is calculated from the averaged profiles of wind and potential temperature profile shown in (a) and (c), respectively. A critical value of $Ri_{c}=0.25$ is indicated by a dashed line in (d) [Colour figure can be viewed at wileyonlinelibrary.com]

Foehn in the mesoscale simulation DX1 is generally characterized by a continuously stably stratified flow up to crest height and beyond (Figures 3a and 4a). The jet‐like wind profile with southerly winds up to about crest level (≈2.5 km AMSL) and westerlies aloft (Figure 4c) indicates the presence of shallow foehn (Mayr and Armi, 2008). The maximum of the southerly foehn jet is about 15 m·s${}^{-1}$ and located close to the surface (Figure 4c). The instantaneous vertical cross‐section of DX1 in Figure 3a depicts hydrostatic gravity waves with a typical backward tilt of the phase lines at three locations at about 1.5 km AGL (at about 4, 13, and 21 km on the abscissa in Figure 3a). However, wave amplitudes and corresponding vertical velocities are rather weak (±1 m·s${}^{-1}$). These waves are not excited by the underlying terrain but rather by the surrounding orography, such as mountain ridges protruding sideways into the Wipp Valley (see Figure 1b). Hence, the wave field in the centre of the Wipp Valley results from three‐dimensional radiation of mountain waves into the cross‐section (e.g., Zängl, 2003; Zängl and Gohm, 2006; Umek *et al*., 2021). It is noteworthy that the overturning of the 292 K isentrope at the Nordkette (at about 24 km on the abscissa in Figure 3a) is caused by flow deflection north of Innsbruck (not shown; Umek *et al*., 2021). During night‐time, mountain waves in DX1 do not break, which is supported by low values of TKE of less than 0.2 m${}^{2}\cdot$s${}^{-2}$ (Figure 4b) and supercritical Richardson numbers (Ri≥0.25; Figure 4d) at about 2 km AMSL where the wave‐induced updrafts are strongest (see above ELL in Figure 3a). In the morning of November 4, when shallow foehn transitions into deep foehn, TKE increases at this height to about 2 m${}^{2}\cdot$s${}^{-2}$ and Ri drops below 0.25, which is an indication of mountain wave breaking in DX1 (not shown).

In contrast to DX1, all LES exhibit overturning isentropes at various locations and scales already during the night (Figure 3b–f). One prominent region of wave breaking in the LES agrees with the location of a small‐amplitude mountain wave above ELL in DX1 (cf. Figure 3a,c). Hence, this wave breaking is most likely terrain induced. However, many of the smaller‐scale eddies cannot be directly attributed to local terrain features and rather originate from K–H instability. This instability results from the strong wind shear at the top of the foehn flow, which is much stronger in the LES than in DX1 (Figure 4c). With increasing grid resolution, K–H instability appears to be more frequent in space and smaller in scale (cf. Figure 3b–f). K–H instability results in turbulent mixing and production of TKE. The latter is illustrated by a TKE peak and a subcritical Richardson number (Ri≤0.25) at about 2 km AMSL in the LESs (Figure 4b,d). Turbulent mixing due to K–H instability strongly modifies the structure of the high‐stability layer capping the foehn jet. This high‐stability layer is first visible as a single layer in the southern part of the Wipp Valley upstream of ELL in essentially all LES (Figure 3b–f) but is completely missing in DX1 (Figure 3a). Near ELL and further downstream, shear‐induced turbulent mixing splits the capping layer into two parts and forms a near‐neutral layer in between (see between 2 and 2.4 km AMSL in Figure 4a). No such splitting and mixing are present in DX1. In the latter, a high‐stability layer is present between about 2.4 and 2.6 km AMSL above ELL in Figure 4a. However, at this particular location, this is caused by a quasi‐stationary mountain wave and only a few kilometres up‐ or downstream of ELL vertical profiles of DX1 are characterized by a rather continuous stratification (cf. Figure 3a).

The K–H instability above the foehn jet, and hence the splitting of the high‐stability layer, is sensitive to the horizontal grid spacing, but more importantly, to the model level spacing. Simulations with a finer vertical resolution exhibit a slightly stronger stratified and shallower upper part at about 2.4 km AMSL than with coarser model level spacings (compare DX40R and DX200R with DX13, DX40, and DX200 in Figure 4a). Additionally, the near‐neutral layer separating the two high‐stability layers is slightly colder. This seems to be partly related to stronger shear‐induced turbulent mixing, especially in the DX40R run (Figure 4b). The LES with the coarsest horizontal and vertical grid, DX200, only marginally resolves K–H waves above the foehn jet (e.g., Figure 3b) with a mean total TKE of only 2 m${}^{2}\cdot$s${}^{-2}$ across the layer capping the foehn jet during the night. In contrast, DX200R better resolves individual K–H waves (Figure 3c) and, hence, produces TKE exceeding 3 m${}^{2}\cdot$s${}^{-2}$ (Figure 4b).

**FIGURE 5:**
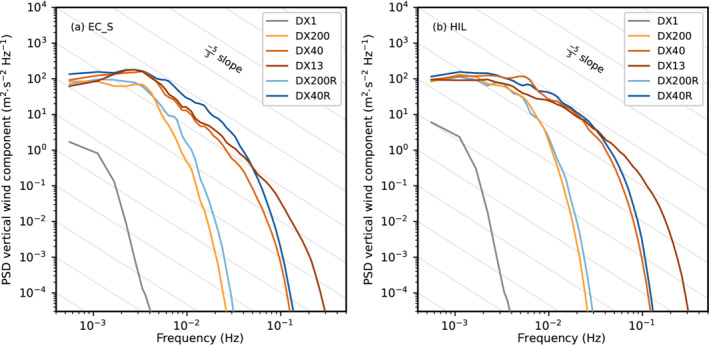
Power spectral density (PSD) of the simulated vertical wind component for different numerical simulations (see legend) calculated for 30‐min periods and averaged between 2230 UTC November 3 and 0600 UTC November 4, 2017, and in the layer from 500 to 1800 m AGL at (a) EC_S and (b) HIL (see Figure 1c for locations). The grey dashed lines indicate the −5/3 slope characteristic of the inertial subrange. The PSD is averaged over 100 equally sized frequency bins in the log${}_{10}$‐space to reduce noise [Colour figure can be viewed at wileyonlinelibrary.com]

Nevertheless, the turbulent eddies resulting from K–H instability are larger in DX200R than in DX40R, with the latter using the same vertical but higher horizontal resolution (cf. Figure 3c,e). Hence, K–H instability is permitted at a horizontal mesh size of 200 m but not resolved at the correct scale. Interestingly, mean total TKE of DX200R, DX40, and DX13 are similar at the level of K–H instability (at about 2 km AMSL in Figure 4b). Nevertheless, DX200R exhibits a considerably larger portion of SGS TKE, whereas DX40 and DX13 are characterized by slightly stronger up‐ and downdrafts (Figures 3c,d,f and 4b). It is noteworthy that the structure of turbulence in terms of eddy size is qualitatively similar in DX13 and DX40 upstream (south) of EC_S but becomes more fine scale in DX13 (i.e., smaller eddies) downstream of EC_S and in the Inn Valley (cf. Figure 3d,f). This similarity in the turbulence structure in the Wipp Valley at EC_S and its difference in the Inn Valley at HIL is supported by a spectral analysis shown in Figure 5. The power spectral density (PSD) of the vertical wind component shown in Figure 5—details on the calculation are given in Umek *et al*. (2021, app. C)—is averaged over all model levels in the layer from 0.5 to 1.8 km AGL above EC_S (about 1.5–2.8 km AMSL). The PSD of DX13 closely follows the PSD of DX40 for frequencies smaller than about $2\times 10^{-2}$ Hz (Figure 5a), which corresponds to periods larger than 50 s. However, the spectra of DX40 and DX13 are less similar in the Inn Valley (Figure 5b). DX40 exhibits a pronounced energy peak at about $6\times 10^{-3}$ Hz (period of about 3 min), which is not present in DX13. Hence, downstream (north) of EC_S turbulence in DX13 is more efficiently transferred to smaller scales compared with DX40. This discrepancy will be analysed in more detail in Section 3.3. This transition to finer‐scale turbulence downstream of EC_S suggests that the fetch from the inflow boundary to develop sufficiently resolved turbulence in DX13 is at least 5 km. Nevertheless, DX13 will still be useful to analyse the impact of the well‐developed turbulence on the CAP in the Inn Valley in the next section.

Finally, reducing the vertical level spacing in DX40R slightly increases the up‐ and downdrafts in the Wipp Valley compared with DX40 (Figure 3d,e) and strongly increases the mean total TKE at ELL from 3 to 6 m${}^{2}\cdot$s${}^{-2}$ (Figure 4b). A similar TKE increase can be identified at EC_S (not shown) and by the spectral analysis in Figure 5a, which shows a higher PSD for DX40R in the frequency range between $5\times 10^{-3}$ and $5\times 10^{-2}$ Hz than for the other LES. DX13 and DX40 share an energy peak at about 2–$4\times 10^{-3}$ Hz. A similar peak is present in DX200 but flattens in DX200R and DX40R. The energy peak illustrates that, with a too coarse vertical resolution, eddies remain too big and do not transfer their energy fast enough to smaller scales. The missing peak at the low‐frequency end of the spectrum at EC_S in DX40R (Figure 5a) and the slightly better alignment with the −5/3 slope indicates that DX40R captures the energy cascade in the inertial subrange up to about $3\times 10^{-2}$ Hz better than the other simulations. However, this is mainly valid only for the high‐stability layer at the top of the foehn flow where the difference in vertical resolution between the simulations is highest (see also Figure 1d). Not surprisingly, DX1 does not capture turbulence and, hence, exhibits generally lower values of PSD than the LES, as well as a rapid drop in the spectrum already at lower frequencies (Figure 5).

### 3.3 Foehn–CAP interaction in the Inn Valley

**FIGURE 6:**
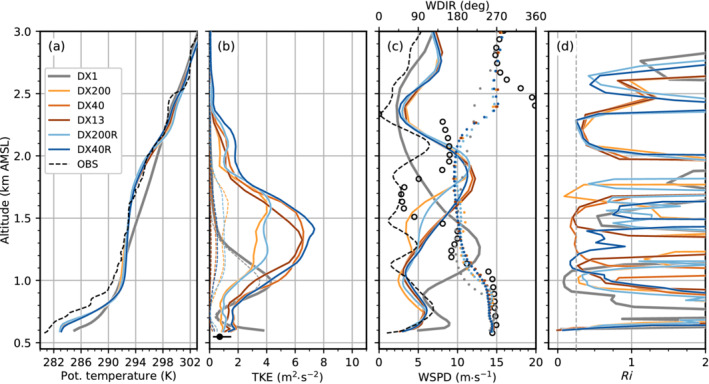
As Figure 4 but for EC_C in the Inn Valley. In addition, observations from a radiosonde launched at Innsbruck Airport about 2 km west of EC_C (IAP in Figure 1c) at 0215 UTC November 4, 2017, are shown in black in (a) and (c). The black dashed line and open circles in (c) denote observed wind speed (WSPD) and wind direction (WDIR), respectively. Moreover, the black dot in (b) indicates the turbulence kinetic energy (TKE) observed at EC_C averaged for the period from 2230 UTC November 3 until 0100 UTC November 4. Observed TKE is calculated based on individual 30‐min intervals, and the horizontal black line illustrates the minimum and maximum values [Colour figure can be viewed at wileyonlinelibrary.com]

When exiting the Wipp Valley, the foehn jet interacts with the CAP in the Inn Valley, which leads to turbulent erosion of the CAP from above (Haid *et al*., 2020; Umek *et al*., 2021). The CAP interaction during the night and the early morning will be the main focus of the following analysis, as the greatest differences between the simulations occur in the second half of this period (Figure 2). Figure 6 shows vertical profiles at EC_C averaged over the first part of the night. Within the CAP, so‐called pre‐foehn westerlies (Umek *et al*., 2021) are present in all simulations, whereas the foehn flow aloft is characterized by southerly winds (Figure 6c). The westerly low‐level jet in the CAP is shallowest but strongest in DX1 and reaches a peak strength of about 9 m·s${}^{-1}$. The simulated jet is deeper and weaker (≈6 m·s${}^{-1}$) in all LES and, hence, in better agreement with observations (Figure 6c). Within the CAP, mean total TKE is between about 1 and 2 m${}^{2}\cdot$s${}^{-2}$ in all LES. At the lowest model level at 10 m AGL, about 65% of the TKE is parametrized in the simulations with Δx=200 m and about 25% with Δx=40 and 13 m. In the mesoscale simulation DX1, mean total TKE at the lowest model level is about 4 m${}^{2}\cdot$s${}^{-2}$ and virtually all is parametrized (Figure 6b). Stronger near‐surface TKE in DX1 results from increased vertical wind shear associated with the stronger and shallower low‐level westerly jet (Figure 6b,c). The higher low‐level TKE in DX1 implies also a stronger (parametrized) turbulent mixing that most likely contributes to faster CAP warming and erosion compared with the LES and the observation (cf. Figure 2). For the period from 2230 UTC November 3 to 0100 UTC November 4, observed TKE is between 0.29 and 1.46 m${}^{2}\cdot$s${}^{-2}$, while the mean observed TKE is 0.71 m${}^{2}\cdot$s${}^{-2}$ (see black dot and horizontal line in Figure 6b) and corresponds well to the TKE simulated in the LES.

The weaker CAP in the Inn Valley in DX1 is also illustrated in Figure 6a. The mean stability in the lowest 400 m AGL in DX1 is lower than in all LES. In the latter, the capping inversion is also slightly elevated from the surface (Figure 6a). However, the CAP in the LES is still too shallow compared with observations (Umek *et al*., 2021). Similar to the Wipp Valley, the stability of the foehn layer above 400 m AGL in DX1 is higher than in the LES (cf. Figures 4a and 6a). In other words, the weaker turbulent mixing in the foehn layer shown in Figure 6b does not lead to a well‐mixed foehn layer in DX1. The temperature and wind profiles of the radiosonde generally agree better with the LES than with DX1 (Figure 6a,c). Larger differences between the observed vertical wind profile and the LES are only present in the layer from about 1.4 to 2.0 km AMSL in Figure 6c. However, some of these discrepancies between simulated and observed profiles can be attributed to the strong heterogeneity of the temperature and wind fields, which results from flow splitting at the Nordkette range north of Innsbruck (Figure 1c) and CAP tilting (Haid *et al*., 2020; Umek *et al*., 2021). For example, flow splitting seems responsible for the weak northeasterly flow encountered by the radiosonde at around 1.6 km AMSL.

Elevated TKE in DX1 is restricted to a relatively shallow layer below the foehn jet and reaches about 4 m${}^{2}\cdot$s${}^{-2}$, of which about 80% is parametrized. In the LES, the peak of the foehn jet is located about 500 m higher than in DX1. Subsequently, a deeper layer of wind shear and turbulent mixing with TKE values between 4 and 7 m${}^{2}\cdot$s${}^{-2}$ depending on the simulation is present in the LES. Interestingly, in the LES as well as DX1, TKE is much higher below than above the respectively simulated foehn jet maxima, which is in agreement with subcritical (supercritical) Ri below (above) the peak of the foehn jet (Figure 6d). Though the temperature profiles in all LES are very similar (Figure 6a), there are some notable differences in the TKE magnitude among the LES (Figure 6b). DX13, DX40, and DX40R reach peak values of 6–7 m${}^{2}\cdot$s${}^{-2}$ in the shear layer of the foehn jet, and more than 90% of this TKE is resolved (Figure 6b). In contrast, maximum TKE in DX200 and DX200R is only about 4 m${}^{2}\cdot$s${}^{-2}$ and about 70–80% is resolved (Figure 6b). These lower values of TKE in DX200 and DX200R are related to reduced wind speed and, more importantly, less vertical wind shear in the layer from 1.3 to 1.8 km AMSL (Figure 6c).

**FIGURE 7:**
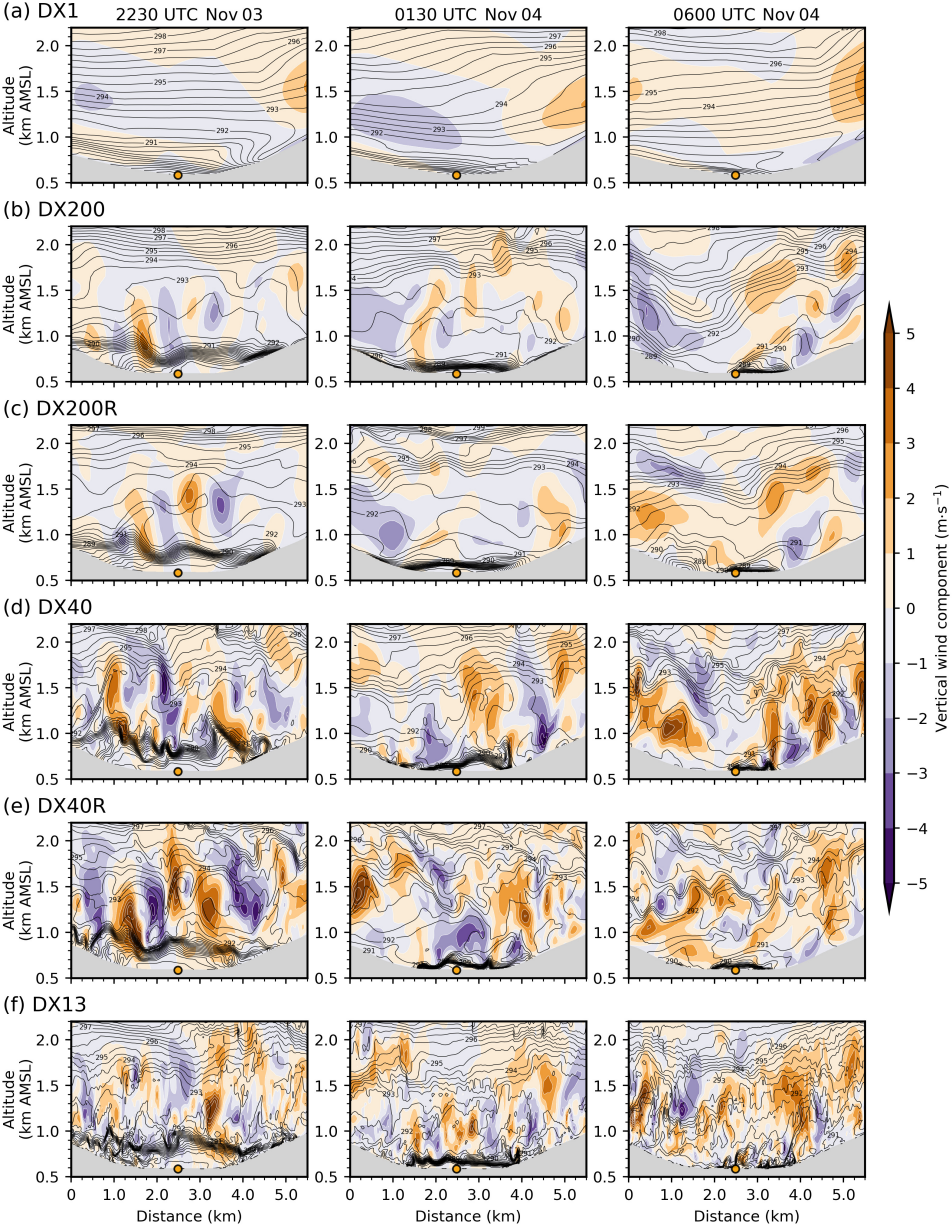
Vertical cross‐section from south to north across the Inn Valley (see transect ${\text{BB}}^{\prime }$ in Figure 1c) of potential temperature and vertical velocity at (left) 2230 UTC November 3, (centre) 0130 UTC November 4, and (right) 0600 UTC November 4, 2017, for (a) DX1, (b) DX200, (c) DX200R, (d) DX40, (e) DX40R, and (f) DX13. Main direction of the flow in the foehn layer above the cold air pool is from left to right. Potential temperature is shown as black contour lines (0.5 K increments). Vertical wind component is illustrated as colour contours with 1 m·s${}^{-1}$ increments. Grey shading shows the model topography, and the orange dot indicates the location of HIL (Figure 1c) [Colour figure can be viewed at wileyonlinelibrary.com]

**FIGURE 8:**
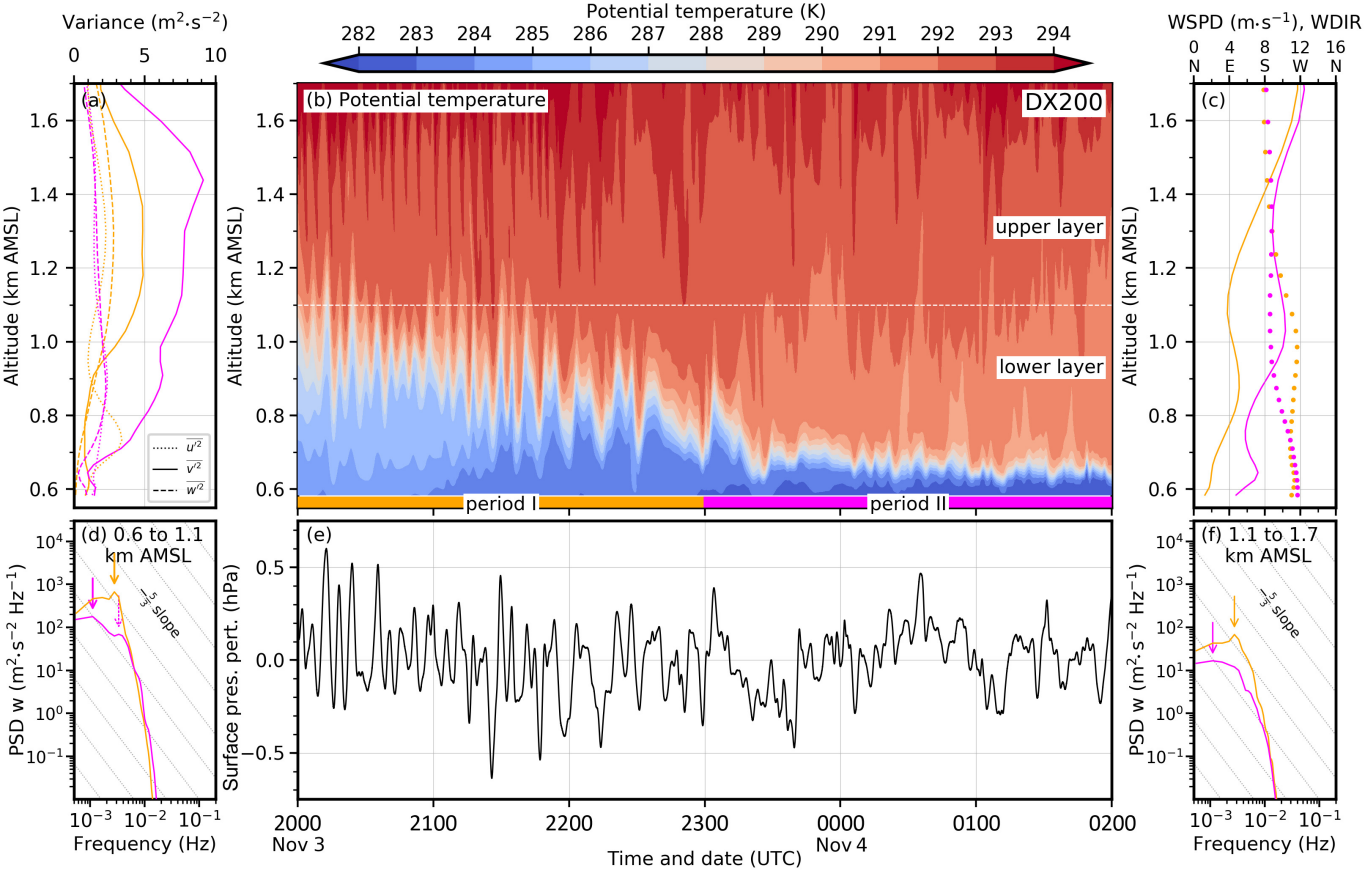
Vertical profile above HIL (Figure 1c) in the simulation DX200, starting from the lowest model level at 10 m AGL. (a) Variance of the west–east (dotted), south–north (solid), and vertical (dashed) velocity component averaged from 2000 to 2300 UTC November 3 (orange, period I) and from 2300 UTC November 3 until 0200 UTC November 4 (magenta, period II). (b) Time–height diagram of potential temperature with periods I and II indicated. (c) Horizontal wind speed (WSPD, lines) and direction (WDIR, dots) averaged for period I (orange) and II (magenta). (d) Power spectral density (PSD) of the vertical wind component (w) in the lower layer from 0.6 to 1.1 km AMSL and averaged for period I (orange) and II (magenta). Peaks of the PSD are indicated by downward‐pointing arrows. Dashed arrows indicate secondary peaks. (e) Surface pressure perturbation at HIL. The time series of pressure has been linearly de‐trended before calculating the perturbations. (f) as (d) but for the upper layer from 1.1 to 1.6 km AMSL [Colour figure can be viewed at wileyonlinelibrary.com]

Figure 7 illustrates the foehn–CAP interaction in more detail for all simulations and three different time steps. The left column depicts the situation in the early night at 2230 UTC November 3. At that time, south foehn has fully established in the Wipp Valley and a 300–400 m deep CAP is simulated in the Inn Valley in all LES (left column in Figure 7b–f). Umek *et al*. (2021, figure 8a) showed that the CAP depth in DX40 is in reasonable agreement with observations for the period from 1900 to 2230 UTC November 3. In DX1, the CAP stability is generally weaker (Figure 7a), which is in agreement with previous statements based on Figure 6a. Isentropes above Innsbruck are nearly horizontal in DX1 and do not show any waves, except for the overturning isentropes close to the Nordkette mountain range caused by flow splitting (left column in Figure 7a). In the LES, however, shear‐flow instability leads to wave‐like structures and eddies at the interface between the CAP and the near‐neutral foehn layer above (Figure 7b–f). For the LES with a horizontal grid spacing of 200 m (Figure 7b,c), relatively large and coherent K–H waves with a wavelength of about 1.5–2 km are present (left column in Figure 7b,c). They are characterized by regular up‐ and downdrafts and partly overturning isentropes. They occur especially in the early night when the CAP is relatively deep. The wavelike structures at the foehn–CAP interface are also depicted by the time–height diagram of potential temperature for DX200 in Figure 8b. During period I (2000 to 2300 UTC) indicated in Figure 8 (orange) the CAP is about 400–500 m deep (see also depth of westerly winds in Figure 8c). A quasi‐periodic vertical displacement of the CAP top by several hundred metres is simulated in period I (Figure 8b). A spectral analysis of the vertical wind component has been performed for this period for two different layers: a lower one representing the CAP (0.6–1.1. km AMSL; Figure 8d) and an upper one representing the foehn (1.1–1.6 km AMSL; Figure 8f). Both spectra show a prominent energy peak at $2.78\times 10^{-3}$ Hz (period of 360 s, orange arrows in Figure 8d,f). With their large vertical amplitude of about 200–500 m and a wavelength of about 1.5–2 km these waves influence a large part of the Inn Valley atmosphere. Based on wavelength and frequency, their apparent phase speed across the valley is 4–5 m·s${}^{-1}$ in DX200. The vertical oscillation in CAP depth leads to pressure perturbations at the surface of about ±0.3 hPa with single maxima of ±0.6 hPa (Figure 8e). It is noteworthy that the averaged velocity variance of the (northerly) v‐component ($\bar{v^{\prime 2}}$) largely exceeds the variance of the (westerly) u‐component ($\bar{u^{\prime 2}}$) and the vertical velocity variance ($\bar{w^{\prime 2}}$) by up to a factor of three (Figure 8a). During period I, the spectral peak is located at a period of 6 min (orange in Figure 8d), and hence the reason of anisotropic variances appears to be submeso motions formed by, e.g., K–H instability. In the later period II, no pronounced spectral peak is present, and this suggests the presence of smaller anisotropic eddies (magenta in Figure 8d,f).

**FIGURE 9:**
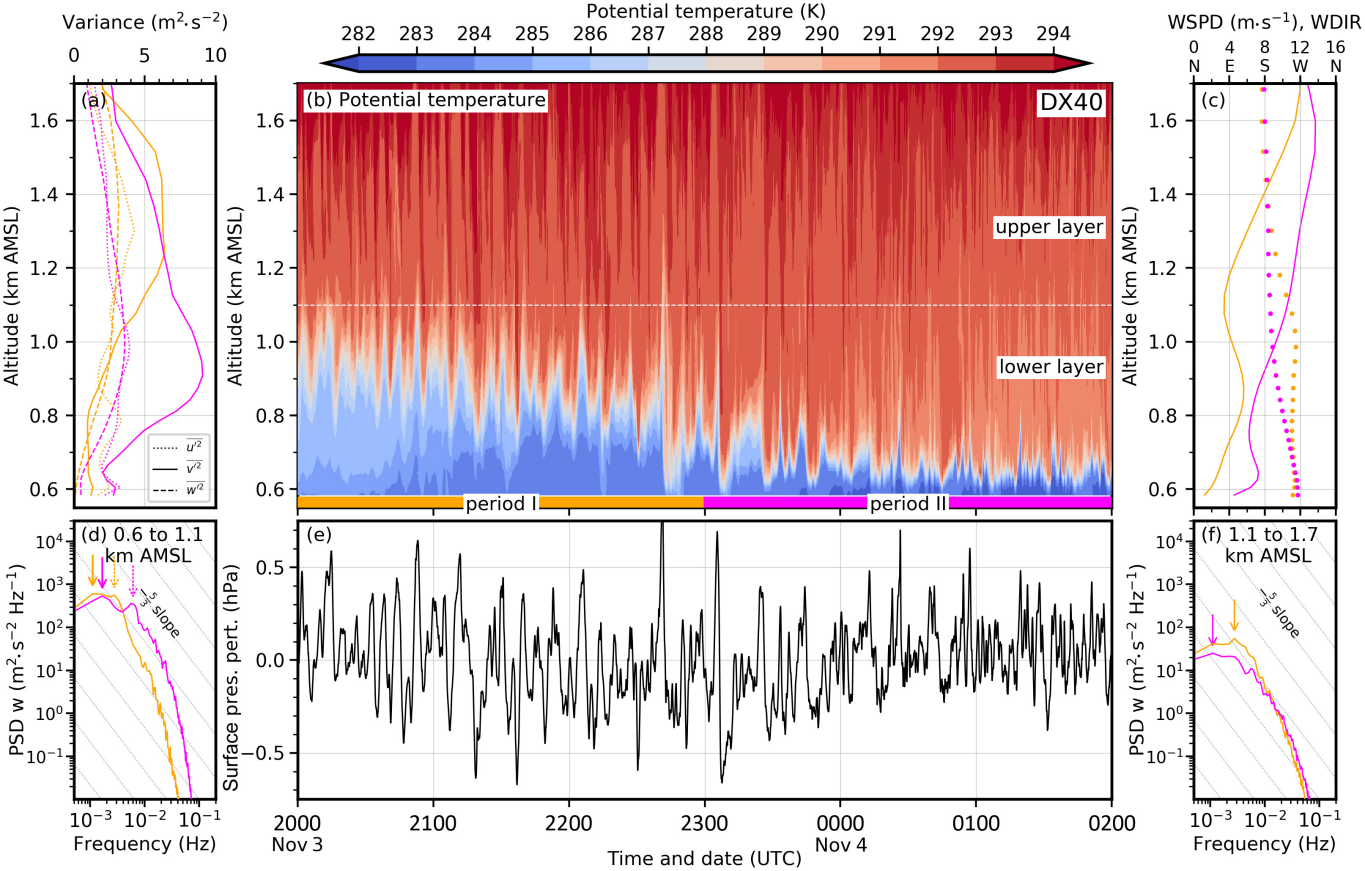
As Figure 8 but for the simulation DX40 [Colour figure can be viewed at wileyonlinelibrary.com]

With a reduced horizontal grid spacing (DX40 and DX40R), individual up‐ and downdrafts related to K–H instability are more vigorous compared with the coarser resolution DX200 and DX200R (cf. Figure 7b–e). This is in line with 50–100% higher TKE (Figure 6b), stronger temperature fluctuations at the foehn–CAP interface (Figure 9b), and associated stronger surface pressure fluctuations (Figure 9e). The magnitude of the latter is about ±0.4 to ±0.8 hPa in period I. Interestingly, DX40 and DX40R still exhibit relatively large K–H waves in the mixed layer between 1.1 and 1.6 km AMSL with a vertical extent and a horizontal wave length comparable to DX200 and DX200R while also smaller‐scale K–H waves start to form at the foehn–CAP interface (Figure 7d,e). Further reducing the horizontal grid spacing in DX13 leads to higher variability and more chaotic flow structure. Hence, individual K–H waves are more difficult to identify visually (Figure 7f). The magnitude of the up‐ and downdrafts remains comparable to the simulations with Δx=40 m (cf., Figure 7d–f).

**FIGURE 10:**
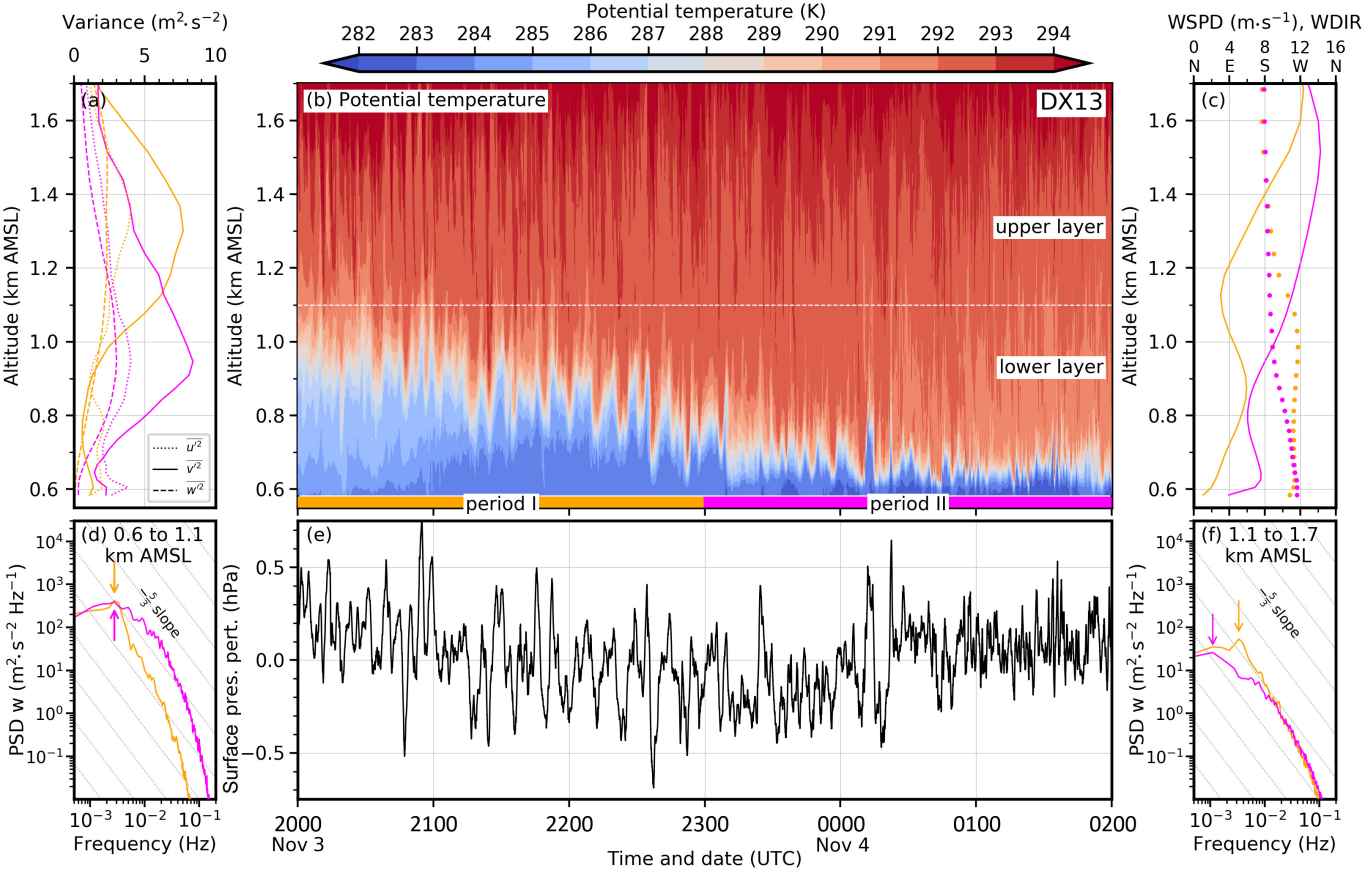
As Figure 8 but for the simulation DX13 [Colour figure can be viewed at wileyonlinelibrary.com]

The PSD of the upper layer of DX40 for period I also exhibits a peak at $2.78\times 10^{-3}$ Hz (orange arrow in Figure 9f). The spectrum of the lower (CAP) layer in DX40 is characterized by a broad peak at low frequencies (orange in Figure 9d). The absolute peak is located at $1.11\times 10^{-3}$ Hz, but a secondary comparable peak can be found again at $2.78\times 10^{-3}$ Hz (solid and dashed orange arrows, respectively, in Figure 9d). The spectra of DX40 and DX13 for period I show some similarities to the spectrum of DX200. For DX13, a prominent peak at $2.78\times 10^{-3}$ Hz is present during period I in both layers at the same frequency as for DX200 mentioned above (orange arrows in Figure 10d,f). Within the CAP, the model is not able to capture the inertial subrange since the spectrum does not follow the $-\frac{5}{3}$ slope (Figure 10d). However, in the upper (foehn) layer the high‐frequency range is partly following the −5/3 slope of the inertial subrange during period I (orange line in Figure 10f). In the upper layer, DX13 captures the inertial subrange to a larger degree compared with DX40 and DX200.

A remarkable change in CAP structure occurs from period I to period II. The latter lasts from 2300 UTC November 3 to 0200 UTC November 4 and is shown in magenta along the abscissa in Figures 8, 9, 10. Turbulent mixing has strongly contributed to CAP erosion which decreased the CAP depth by about 50% (cf. period I and II in Figures 8, 10). However, CAP erosion during the night is too strong compared with observations (Umek *et al*., 2021). Simulated pre‐foehn westerlies are on average only present up to 200 m AGL (about 0.8 km AMSL, solid magenta line in Figures 8c, 9c, and 10c). Additionally, the stability of the CAP has intensified. Despite the differences in the small‐scale structure, bulk properties such as the mean CAP depth and stability are remarkably similar in all LES (cf. Figure 6a, middle column of Figure 7b–f, and period II in Figures 8, 10). Nevertheless, the higher spatio‐temporal variability with increasing resolution implies strong differences at the local scale. For example, at 0130 UTC November 4 the CAP is relatively homogeneous across the Inn Valley in DX200 and DX200R but strongly deformed in the LES with higher resolution (middle column in Figure 7). In the latter, some of the smaller‐scale eddies are almost able to penetrate to the surface, which leads in period II to stronger low‐level temperature fluctuations in DX40 and DX13 (Figures 9b and 10b) than in DX200 (Figure 8b). Until 0600 UTC November 4, the turbulent CAP erosion is further ongoing (right column in Figure 7). In the LES with a horizontal grid spacing of Δx=200 and 40 m, the remaining CAP is represented by a dome of colder air in the middle of the Inn Valley (right column in Figure 7b–e), whereas it is much more broken up into individual patches of colder air in DX13 (right column in Figure 7f). This higher spatio‐temporal variability of the CAP in DX13 can also be seen in the larger fluctuations of the near‐surface potential temperature towards the end of the night shown in Figure 2. Haid *et al*. (2022) also state that the temperature field along the Inn Valley becomes increasingly heterogeneous with ongoing CAP erosion during several PIANO IOPs.

In comparison with the earlier period I, the spectra of the vertical wind component during period II exhibit reduced magnitudes in the low‐frequency range and no pronounced peak for DX200 (solid magenta line in Figure 8d,f). Only a secondary, less pronounced “bump” in the spectrum can be found in the CAP layer at about the same frequency as the main peak during period I (cf. dashed magenta and solid orange arrow in Figure 8d). This suggest that eddies of a similar size as in period I are influencing the CAP, but are less vigorous. The distance between individual maxima and minima of vertical velocity in Figure 7b,c is comparable at all three times and, therefore, also suggests a similar wavelength of the resolved eddies during the night. Overall, spectra for period II of DX40 and DX13 also show less isolated peaks in the submeso range but at the same time more energetic turbulence in the high‐frequency range in the lower layer compared to period I (cf. Figures 9d,f and 10e,f). Both factors contribute to the fact that the spectra are closer to the $-\frac{5}{3}$ slope and, hence, cover part of the inertial subrange. However, there is one notable deviation from a smooth spectrum, which is an isolated peak at $6.11\times 10^{-3}$ Hz (period of about 160 s) in the shallow CAP layer of DX40 (dashed magenta arrow in Figure 9d). This isolated peak in the spectrum points to too much energy accumulation at this scale compared with DX13 (cf. magenta line in Figures 9d and 10d). Hence, the energy cascade towards smaller eddies following Kolmogorov's inertial subrange appears to be not fully captured by DX40 in the foehn–CAP interaction zone. During period II the maxima of the individual velocity variances can be found at lower altitudes compared with period I (Figures 8a, 9a, and 10a), which is consistent with a shallower CAP and, hence, a lower foehn–CAP interaction zone (Figures 8b, 9b, and 10b). Additionally, anisotropy of turbulence increases during the night in DX200 and DX40 as $\bar{v^{\prime 2}}$ increases more than the variance of the other components (Figures 8a and 9a). However, this increase in anisotropy in time does not occur in DX13 (Figure 10a). This raises the question of whether the anisotropy is overestimated for coarser horizontal resolutions.

Over the Inn Valley, the effect of increased vertical resolution (cf. Figure 1d) on K–H instability and shear‐driven turbulence at lower levels is rather limited (Figure 5b), which is in contrast to the effect on K–H instability at higher levels in the Wipp Valley (Figure 5a and Section 3.2). In line with this, no strong impact of the refined model level spacing on the mean total TKE can be detected in the Inn Valley above the CAP (Figure 6b).

## 4 DISCUSSION

In Section 3 and Umek *et al*. (2021) we showed that LES are able to represent processes of foehn–CAP interaction in the Inn Valley more explicitly and more realistically than mesoscale simulations. However, the differences among the LES with different horizontal and vertical resolutions are less pronounced for the mean structure of the foehn and the CAP (e.g., Figures 4a,c and 6a,c) as well as for the overall timing of the event (e.g., Figure 2). Nevertheless, higher horizontal and/or vertical resolution leads to a better representation of turbulent processes in the numerical simulations. This also seems true for the resolved eddy size (Figures 3 and 7), as shown in the associated turbulence spectra (Figures 5, 8,f, 9d,f, and 10d,f).

**FIGURE 11:**
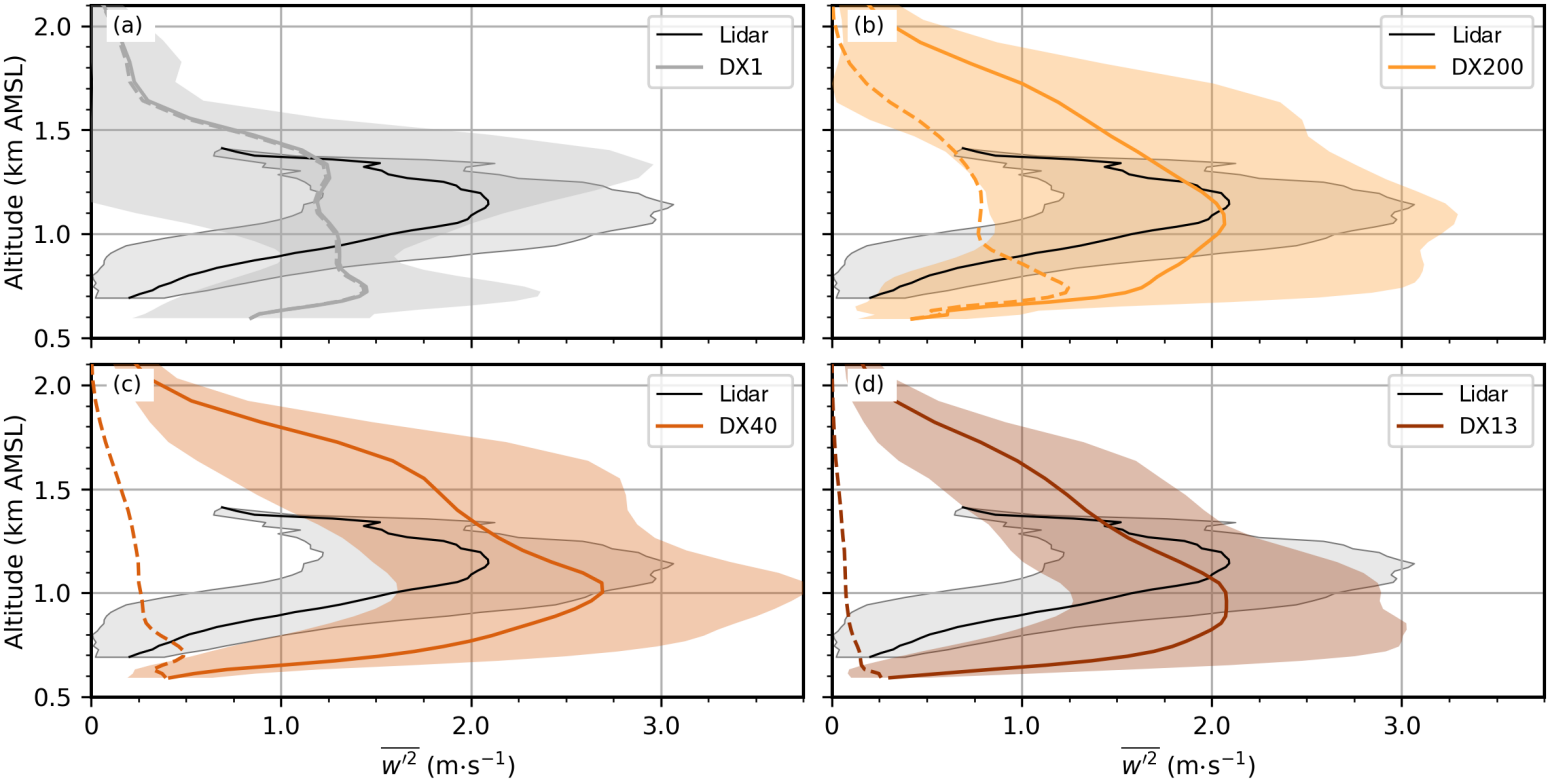
Averaged vertical velocity variance $\bar{w^{\prime 2}}$ calculated from Doppler wind lidar observations (black line) and WRF simulations (grey and colour lines) of (a) DX1, (b) DX200, (c) DX40, and (d) DX13 at lidar site SL74 (Figure 1c) for the period from 2000 UTC November 3 to 0600 UTC November 4, 2017. Averaged simulated total (resolved‐scale plus SGS) variance is shown as solid lines and averaged SGS contribution as a dashed line. Averaged variances are based on individual vertical velocity variance profiles derived from 18‐min long periods of vertical lidar measurements conducted once an hour and individual variance profiles calculated for 30‐min long time windows from WRF data. For DX1, individual SGS velocity variances were not available and $\bar{w^{\prime 2}}$ is approximated using TKE profiles and assuming isotropic turbulence (i.e., $\bar{w^{\prime 2}}=(2/3)\text{TKE}$). Shaded areas denote the standard deviation of total $\bar{w^{\prime 2}}$ for the whole 10‐hr period [Colour figure can be viewed at wileyonlinelibrary.com]

Further support for the statement of more realistic turbulence with increasing model resolution comes from a comparison with observed vertical velocity variances. Two Doppler wind lidars located near the centre and in the south of Innsbruck (SL74 and SL75 in Figure 1c) performed vertical velocity measurements during 18‐min time windows once an hour (Haid *et al*., 2020). These lidar observations are used to derive the vertical velocity variance $\bar{w^{\prime 2}}$, which represents one component of TKE. Figure 11 shows $\bar{w^{\prime 2}}$ averaged during the night from 2000 UTC November 3 until 0600 UTC November 4 at the location of the SL74 lidar, close to the city centre. The profiles are very similar at the second location SL75 (not shown). First of all, it is apparent that the averaged $\bar{w^{\prime 2}}$ in DX1 does not reproduce the jet‐like structure as seen in the observations or the LES (Figure 11). Furthermore, it underestimates the elevated peak by about 40% and overestimates the variance near the surface. This overestimation of near‐surface turbulence in DX1 has also been shown for TKE at EC_C a few kilometres further west (cf. Figure 6b). Maximum values of simulated mean total (resolved‐scale plus SGS) $\bar{w^{\prime 2}}$ in DX200 and DX13 are comparable to averaged lidar‐derived variances (solid lines in Figure 11b,d), whereas simulated mean total $\bar{w^{\prime 2}}$ in DX40 is too large compared with observations (Figure 11c). In DX200, a considerable part of the mean total variance is coming from the SGS contribution. In contrast, in DX13, the vast majority of $\bar{w^{\prime 2}}$ is explicitly resolved, as SGS $\bar{w^{\prime 2}}$ is low except near the surface (cf. dashed lines in Figure 11b,d). Moreover, the standard deviation of the simulated total $\bar{w^{\prime 2}}$ overall decreases with decreasing resolution from DX13 over DX40 to DX200 (Figure 11). This seems to be caused by larger, more vigorous eddies in the coarser LES. Interestingly, near‐surface $\bar{w^{\prime 2}}$ is also overestimated in DX200, but DX40 and D13 are in better agreement with observations (Figure 11).

In particular, total TKE (resolved‐scale plus SGS) produced by K–H instability exhibits strong differences of up to 100% between “true” LES resolution (e.g., Δx=13.33 m), which captures at least part of the inertial subrange, and hectometre resolution (e.g., Δx=200 m, Figures 4b and 6b), which lies in the “grey zone” of turbulence (e.g., Cuxart,, 2015). For turbulence generated at the foehn–CAP interface in the Inn Valley, the strongest increase in TKE and vertical velocity variance was found when decreasing the horizontal grid spacing from 200 m to 40 m (Figures 6b and 11). Further decreasing the horizontal mesh size to 13.33 m slightly reduced TKE in the Inn Valley and vertical velocity variances (Figures 6b and 11), whereas increasing the vertical resolution had almost no effect regarding TKE in the CAP (Figure 6b). In contrast, for the turbulence generated at the top of the foehn flow in the Wipp Valley, stronger TKE was found for higher vertical resolution independent of horizontal resolution. In other words, the magnitude of TKE can be sensitive to both horizontal and vertical resolution. Which of these is most important, however, depends on the flow regime and location. Owing to numerical instabilities, no vertical refinement was possible for the simulation with a horizontal grid spacing of 13.33 m without tremendously increasing computational costs by strongly reducing the time step in the innermost LES domain. This is unfortunate, as doubling the vertical resolution in this simulation would have led to nearly isotropic grid cells at the foehn–CAP interface. We suggest that future work should investigate the influence of an isotropic grid on turbulence at the foehn–CAP interface.

In the Wipp Valley, the combination of a sufficiently small horizontal and vertical grid spacing is important. Whereas the mesoscale simulation DX1 only exhibits low‐amplitude hydrostatic mountain waves over the Wipp Valley, the LES produces K–H instability that leads to TKE production and the splitting of the high‐stability layer on top of the foehn jet. This difference in flow response between the mesoscale and the microscale simulations recalls the controversy between Afanasyev and Peltier (2001a; 2001b) and Farmer and Armi (1999, 2001). On the role of mountain wave breaking and shear‐induced small‐scale instabilities. Whereas Afanasyev and Peltier (2001a; 2001b) simulated a shooting flow with mixed conditions aloft in response to large‐amplitude mountain wave breaking, Farmer and Armi (1999, 2001) Showed with their observations that rather small‐scale instabilities are important during the phase of the flow establishment. In their work, K–H instability is important for turbulent mixing and the generation of a mixed layer on top of the shooting flow. In agreement with these controversial results, our mesoscale simulation tends to favour mountain wave breaking, since it neither resolves nor correctly parametrizes K–H instability. The LES suggests that, in reality, a combination of both mountain wave breaking and K–H instability is possible. However, the latter can also occur without the former.

K–H waves are part of so‐called submeso motions that can contribute to the gustiness of foehn, or a downslope wind storm in general. For example, Belušić *et al*. (2007) found a period of 3–11 mins for pulsations of the Croatian bora. Tollinger *et al*. (2019) detected pulsations with periods between 7 and 16 min in their mesoscale simulations of a downslope wind storm in Greenland. However, with their 1 km mesh size they were not able to fully resolve K–H waves. This period is comparable to the peak at $2.78\times 10^{-3}$ Hz (period of 6 min) thath can be found in most of the spectra shown in Figures 8, 9, and 10 as well as the secondary peak at $6.11\times 10^{-3}$ Hz (dashed magenta arrow in Figure 9d). Also Afanasyev and Peltier (1998) mention that pulsations due to K–H instability can cause transience in the fully developed downslope wind storm. The CAP in our simulations is influenced by transient motions caused by K–H waves, which cause vertical and horizontal deformations of the CAP at various length scales. This also leads to intermittent phases when foehn air can almost penetrate to the valley floor (Figures 8b, 9b, 10b), resulting in transient warming and gustiness at the surface.

Not surprisingly, the relative proportions of resolved‐scale and SGS TKE depends on the grid resolution. More surprising are low values of total TKE in the mesoscale simulation near the top of the foehn flow and at the foehn–CAP interface (Figures 4b and 6b). In other words, the PBL parametrization does not correctly capture the SGS effect of K–H instability in complex terrain. We did not investigate whether neglecting lateral shear in the one‐dimensional PBL parametrization of the mesoscale simulation DX1 has contributed to this model deficiency. Such a hypothesis was already raised by Zängl *et al*. (2004). Hence, a more systematic study of the effect of lateral shear should be done in the future, as it also proved relevant for thermally driven flows in complex terrain (e.g., Goger *et al*., 2018).

The spectral analysis showed that DX200 does not capture the inertial subrange, neither in the Wipp Valley nor in the Inn Valley (cf. Figures 5 and 8d,f). In contrast, with a horizontal grid spacing of Δx=40 m the inertial subrange is partly covered in the mixed layer above the CAP (1.1–1.6 km AMSL in Figure 9f). During period I, when the lower layer from 0.6 to 1.1 km AMSL is mainly characterized by the presence of the CAP, no inertial subrange is visible in the spectral analysis (orange line in Figure 9d). Later, in period II, when only a very shallow CAP is left after ongoing turbulent erosion, parts of the inertial subrange are resolved but a pronounced peak in the spectrum suggests that the energy transport towards smaller scales is not fully captured (Figure 9d). Individual eddies at the foehn–CAP interface in DX40 are among the most vigorous of all simulations. This is also indicated by the largest mean total $\bar{w^{\prime 2}}$ in the layer of foehn–CAP interaction (Figure 11). Additionally, the vertical CAP displacement and, subsequently, the surface pressure perturbations are strongest in DX40 (cf. Figure 9b,e). Further increasing the horizontal resolution in DX13 smooths the spectrum in the CAP for the later period II (Figure 10d). In other words, decreasing grid spacing from hectometre to several decametres can become counterproductive as it may lead to too much energy on the smallest resolvable scale that then might produce the too strong CAP erosion. Hence, an even higher (sub‐decametre) resolution may be needed in such situations to foster energy transfer to smaller scales. In period I, however, the spectra of DX40 and DX13 are very similar (orange in Figures 9d and 10d), with a pronounced peak at lower frequencies and no secondary peaks in the high‐frequency range. It would be interesting to investigate the performance of a simulation with a horizontal grid spacing of about 10 m and an even more refined level spacing, optimally achieving a grid aspect ratio close to one in the foehn–CAP interaction zone (Zhong and Chow, 2013). It is likely that the breakdown of eddies to smaller scales is represented more realistically in such a simulation. This could also delay CAP erosion and improve the timing of the simulated foehn breakthrough. It may also be the case that the degree of anisotropy would be different in such a simulation. However, owing to the enormous computational costs, such a simulation was not possible in this study.

**FIGURE 12:**
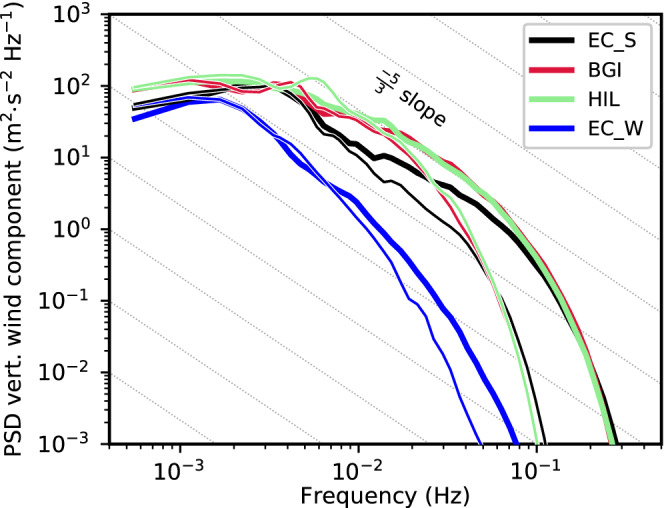
Power spectral density (PSD) of the simulated vertical wind component (w) for DX13 (thick lines) and DX40 (thin lines) at different locations in Inn Valley and Wipp Valley (see legend and Figure 1c) based on 30‐min periods and averaged over the layer 0–1800 m AGL and between 2230 UTC November 3 and 0600 UTC November 4, 2017. The grey dashed lines indicate the −5/3 slope characteristic of the inertial subrange. The PSD is averaged over 100 equally sized frequency bins in the log${}_{10}$‐space to reduce noise [Colour figure can be viewed at wileyonlinelibrary.com]

In Section 3.2 we noted that turbulence characteristics, including spectra, are similar in DX13 and DX40 in the Wipp Valley. We speculated that turbulence has not fully developed in this part of the DX13 domain as we did not use a cell perturbation method to spin up turbulence at the inflow boundary (e.g., Mirocha *et al*., 2014; Muñoz‐Esparza *et al*., 2014). In order to further explore this hypothesis, we performed additional spectral analyses for several grid points along the foehn jet from the Wipp Valley northward (downstream) towards the Inn Valley. For DX13, the PSD at ELL (not shown) exhibits an overall weaker magnitude than at EC_S. At EC_S, the low‐frequency range is slightly more pronounced and, hence, bigger eddies seem to be present at this location (Figure 12). At the Wipp Valley exit (BGI in Figure 1c), an energy shift towards higher frequencies takes place in DX13 (cf. solid lines for EC_S and BGI and $\geq 6\times 10^{-3}$ in Figure 12). Therefore, for DX13, the energy cascade towards smaller scales is better reproduced at BGI than at EC_S (Figure 12). This is also valid further downstream at HIL in the Inn Valley, where the PSD is comparable to the one at BGI in DX13 (solid lines in Figure 12). Hence, we conclude that turbulence in DX13 needs a fetch of about 6–8 km downstream of the domain boundaries to become fully developed, and this is the case north of EC_S. This problem seems to affect only DX13, as the domain boundaries of DX40 and DX200 are located more then 30 km south of our area of interest (Figure 1b). Finally, west of Innsbruck, where a stronger CAP is present (Umek *et al*., 2021) and interaction with the foehn is less vigorous, DX13 is only able to capture a small part of the inertial subrange for frequencies between $2\times 10^{-3}$ and 10${}^{-2}$ Hz (EC_W in Figure 12). In contrast, DX40 exhibits a slightly stronger drop of the PSD in this frequency range (Figure 12). However, differences between the PSD of DX40 and DX13 are smaller at EC_W than at other locations where the foehn–CAP interaction is stronger. Though the increased horizontal resolution of DX13 slightly increases the amount of resolved turbulence in the CAP, a sub‐decametre horizontal grid spacing seems to be necessary to fully capture dynamics in the lower part of the CAP (Cuxart, 2015).

## 5 CONCLUSIONS

The WRF model was used in mesoscale and LES mode to study a south foehn event in the Inn and Wipp valleys, Austria, which occurred during PIANO IOP2. This study extends the work of Umek *et al*. (2021) and evaluates the influence of the horizontal and vertical grid resolution on the simulated foehn jet in the Wipp Valley and its interaction with a CAP in the Inn Valley during night‐time. Simulations were performed with four different horizontal mesh sizes (1 km, 200, 40, and 13.33 m) and two different vertical resolutions based on 80 and 110 model levels. The main conclusions are as follows.

- The mesoscale simulation with a horizontal grid spacing of Δx=1 km fails to capture the evolution of the nocturnal CAP in the Inn Valley as well as the stratification and depth of the foehn flow in the Wipp Valley. More specifically, CAP erosion and foehn breakthrough in the Inn Valley occur too early. The foehn layer in the Wipp Valley is too shallow, too stable, and too cold near the surface due to insufficient turbulent mixing inside and above the foehn jet, with TKE being virtually zero near the top of the foehn flow. The reason is that the mesoscale model does not capture shear‐induced K–H instability above the foehn jet and at the foehn–CAP interface, which appears to be the dominant process of TKE production during the foehn phase investigated. The mesoscale model only simulates low‐amplitude non‐breaking hydrostatic mountain waves. The cold bias in the foehn flow, the warm bias in the CAP, and the resulting too early foehn breakthrough is in line with previous studies and points to a systematic misrepresentation of turbulent processes over complex terrain by current one‐dimensional PBL parametrizations.
- All LES are able to capture the stability and wind profile of the foehn in the Wipp Valley and the CAP formation in the Inn Valley during the early night. CAP erosion is delayed compared with the mesoscale simulation but still occurs too early compared with the observations, regardless of the horizontal and vertical model resolution. Surprisingly, LES grid resolution has only little influence on the mean flow characteristics despite a factor of 15 difference between the coarsest and the finest horizontal mesh size. In other words, even with Δx=200 m the model is able to form a well‐mixed foehn layer in the Wipp Valley with a capping high‐stability layer that splits into two parts due to turbulent mixing resulting from K–H instability. Also, the depth and the stability of the CAP in the Inn Valley and the time of the foehn breakthrough are very similar in all LES.
- However, differences between the LES can clearly be found in terms of turbulence characteristics. A horizontal mesh size of Δx=200 m permits K–H instability but does not fully resolve associated K–H waves. Hence, it leads to higher TKE compared with the mesoscale simulation but still total (resolved‐scale plus SGS) TKE is considerably smaller compared with higher resolution LESs (e.g., only about half at the top of the foehn jet and at the foehn–CAP interface). Surprisingly, increasing the vertical resolution for the simulation with Δx=200 m by about halving the level spacing results in a very similar TKE profile in the Wipp Valley as when the horizontal mesh size is decreased from Δx=200 m to 40 m without changing the vertical resolution. The former approach is not perfect as it increases the anisotropy of the grid, but it is much less computationally expensive. The benefit is a more realistic spatio‐temporal variability in foehn and CAP structure caused by K–H instability, which is one important type of submeso motion that contributes to TKE production.
- Spectral analysis of the vertical velocity perturbations indicates that the inertial subrange is insufficiently resolved at Δx=200 m and 40 m, and an accumulation of energy occurs in the short‐wave part of the resolvable scale. In particular, with Δx=40 m it appears that the eddy size stays too large due to insufficient energy cascading to smaller scales. As a consequence, turbulent eddies are too vigorous compared with Δx=13.33 m and are able to penetrate through the CAP to the surface, resulting in transient warming. In line with this, the run with Δx=40 m exhibits the largest values of vertical velocity variances inside the CAP and at the foehn–CAP interface.
- The reason for not being able to capture the right timing of the foehn breakthrough even with Δx=13.33 m could not be found. It is likely that for shallow cold pools with depths between about 100 and 300 m the vertical resolution is not high enough to resolve the dominant eddy size at the foehn–CAP interface. It is also likely that the chosen decametre resolution in both horizontal and vertical directions is simply insufficient to resolve the turbulent processes inside the stably stratified and less turbulent CAP.
- To improve high‐resolution NWP in complex terrain, we suggest to explore the benefit of increasing the model resolution from 𝒪(1 km) to 𝒪(100 m), with Δx of about 200–500 m being realistic in the near future, together with increasing vertical resolution in the boundary layer. Our results suggest a clear benefit despite the fact that such a horizontal resolution lies in the “grey zone” of turbulence. We argue that 𝒪(100 m) permits (although not completely resolves) shear‐flow instability, which is an important process of turbulence generation in complex terrain. Hence, the argument is similar to the introduction of convection‐permitting models with 𝒪(1 km) more than a decade ago with the aim to improve quantitative precipitation forecasts (e.g., Clark *et al*., 2016), despite the fact that structural convergence is not achieved at that resolution (e.g., Panosetti *et al*., 2019). With 𝒪(100 m), shear‐flow instability does not need to be fully parametrized by the PBL scheme. However, the PBL scheme would still have to be adapted to efficiently transfer the resolved turbulence to smaller scales and to parametrize the smaller scale turbulence by not only accounting for vertical but also for horizontal mixing in a three‐dimensional manner.

Hence, future work should focus on assessing the benefit of three‐dimensional scale‐aware PBL parametrizations in the realistic future NWP mesh size range of 200 m to 1 km for improving NWP in complex terrain (Doubrawa and Muñoz‐Esparza, 2020). Such an effort should not only focus on foehn and CAPs, but also on other types of local and regional wind systems, including valley and slope winds. It is expected that a better representation of such phenomena will improve transport and exchange processes over complex terrain and NWP in general. Such a study would be in line with the main goals of the TEAMx programme and experiment (Serafin *et al*., 2020).
